# Effects of dialysis modality choice on the survival of end-stage renal disease patients in southern China: a retrospective cohort study

**DOI:** 10.1186/s12882-020-02070-7

**Published:** 2020-09-24

**Authors:** Zhiren He, Haijing Hou, Difei Zhang, Yenan Mo, La Zhang, Guobin Su, Junjie Lin, Liming Lu, Jingyao Huang, Yewen Gu, Ying Zhang, Jingxia Lin, Fengling Yuan, Yu Peng, Hui Liang, Daixin Zhao, Fuhua Lu, Xusheng Liu, Lixin Wang

**Affiliations:** 1 Department of Nephrology, The Second Affiliated Hospital of Guangzhou University of Chinese Medicine (Guangdong Provincial Hospital of Chinese Medicine), No. 111 Dade Road, Guangzhou, 510120 Guangdong China; 2 The Second Clinical College of Guangzhou University of Chinese Medicine, Guangzhou, Guangdong China; 3 Clinical Research and Data Center, South China Research Center for Acupuncture and Moxibustion, Medical College of Acu-Moxi and Rehabilitation, Guangzhou University of Chinese Medicine, Guangzhou, Guangdong China; 4 The First Affiliated Hospital of Guangzhou University of Chinese Medicine, Guangzhou, Guangdong China

**Keywords:** Peritoneal dialysis, Hemodialysis, Age, End-stage renal disease, All-cause mortality

## Abstract

**Background:**

The optimal choice of treatment, with hemodialysis (HD) or peritoneal dialysis (PD), for end-stage renal disease (ESRD) patients, is still controversial. Only a few studies comparing HD and PD have been conducted in China, which has the largest number of dialysis patients in the world.

**Methods:**

A retrospective cohort study was conducted on ESRD patients who began renal replacement treatment from January 1, 2012 to December 31, 2017 in Guangdong Provincial Hospital of Chinese Medicine. Propensity scoring match was applied to balance the baseline conditions and multivariate Cox regression analysis to compare the mortality between HD and PD patients, and evaluated the correlation between mortality and various baseline characteristics.

**Results:**

A total of 436 HD patients and 501 PD patients were included in this study, and PD patients had better survival than HD patients, but the difference was not statistically significant. For younger ESRD patients (≤60-year-old), the overall survival of PD was better than that of HD, but HD was associated with a lower risk of death in older patients (> 70-year-old). This difference was still significant after adjustment for a variety of confounding factors. Female gender, age at dialysis initiation, cardiovascular disease, cholesterol, and HD were risk factors of all-cause mortality in the younger subgroup, while PD was risk factor in the older subgroup.

**Conclusion:**

PD may be a better choice for younger ESRD patients, and HD for the older patients.

## Background

China has a large territory, a huge population, and insufficient medical resources. The number of end-stage renal disease (ESRD) patients is continually rising. Hemodialysis (HD) is currently the main method of renal replacement therapy, and the number of peritoneal dialysis (PD) patients has increased rapidly [1]. The prevalence of HD and PD is estimated to be 402.18 and 39.95 per million individuals in China, and the corresponding number was approximately 553,000 HD patients and 55,000 PD patients in 2015 [2].

Whether HD or PD is a better choice for dialysis patients is still controversial [3]. Survival outcomes are major indicators for evaluating the efficacy and effectiveness of these two dialysis modalities. The impact of HD and PD on patient survival is conducted via randomized controlled trial (RCT), which is not commonly performed in dialysis patients because they developed a strong preference for a specific dialysis modality while being educated about the treatment. Only one RCT was stopped prematurely due to the low inclusion rate [4]. Several observational studies have compared PD and HD and attained conflicting results. Studies from Norway [5] and Denmark [6] have reported improved survival in PD patients, while some studies from Korea [7] and Singapore [8] have pointed to a survival benefit for HD patients. Nonetheless, there no evidence for a difference between both modalities in studies from Canada [9, 10], USA [11], and Taiwan [12]. Recently, a single-center retrospective study from Northern China showed a similar effect of HD and PD [13]. However, this study had limitations of small sample size and short duration of follow-up. Moreover, factors including climate condition, living habits, and economic resources vary across different regions in China, and might affect the survival outcomes of HD and PD patients. Therefore, the actual effect of HD and PD on survival is yet to be elucidated [3].

In order to compare the survival outcomes of patients using different dialysis modalities, a retrospective cohort study was conducted on ESRD patients who needed renal replacement treatment in Guangdong Provincial Hospital of Chinese Medicine in Southern China. Herein, we presented the results of the comparison of mortality between HD and PD patients and evaluated the correlation between mortality and various baseline covariates.

## Methods

A retrospective cohort study was conducted from January 1, 2012 to December 31, 2017 in Guangdong Provincial Hospital of Chinese Medicine Nephrology Department. The inclusion criterion was this period judged by the clinician during which long-term maintenance of renal replacement therapy was needed.

The outcome events were determined from previous follow-up records. The main outcome was all-cause mortality of the patient. The censor event included the patient switching to another dialysis mode or undergoing a kidney transplant or transfer to another dialysis center to continue treatment or reach the end of follow-up (December 31, 2018). The HD patients needed to return to the dialysis center for dialysis treatment 2–3 times a week; hence, the event could be recorded with a date. The majority of the PD patients were treated with continuous ambulatory peritoneal dialysis (CAPD) and needed to revisit every month for therapeutics. Therefore, follow-up visits were scheduled every 1–3 months, and the exact occurrences of the events were clarified by interviewing family members.

Baseline demographics, comorbid conditions, and laboratory test results were obtained by reviewing the electronic medical records. Demographic data included the date of birth, gender, the start of dialysis, the primary onset of kidney, height, and weight at the start of dialysis. The comorbidities were identified at the baseline by the International Classification of Diseases, 9th and 10th Revision (ICD-9 and ICD-10) codes, and the Charlson comorbidities index (CCI) was calculated based on Quan et al’s method [14]. Cardiovascular diseases include asymptomatic myocardial ischemia (occult coronary heart disease), angina pectoris, myocardial infarction, ischemic heart failure (ischemic heart disease).

Laboratory indicators included hemoglobin, plasma albumin, serum creatinine, triglyceride, and cholesterol. These items were considered potential predictors or confounders.

Since this was a retrospective analysis, selection bias towards eligible patients causes an imbalance in the baseline status. Therefore, in the analysis, we used propensity score matching (PSM) to reduce the effect of selectivity bias. Furthermore, medical records were collected to deduce the statistical significance of the results. Based on the mortality rates reported in previous studies [8] in Singapore, the 5-year mortality difference between the two dialysis methods was 6.4% (PD: 13.4% vs. HD: 7.0%); α = 0.05 and β = 0.10. Using Pearson’s chi-square test, the estimated sample sizes for a two-sample proportion is 872 cases, or 436 cases for each group.

Baseline characteristics and laboratory tests of HD patients were compared to those of PD patients. The normally distributed continuous variables were presented as mean ± standard deviation (SD), and t-tests were used for comparison. Skewed data were presented as median and rank, and Mann–Whitney U test was used for comparison between groups. Missing data were filled in using a multiple imputation by chained equations algorithm by using R’s *MICE* package. The filling method is “mean”. The categorical variables were presented as percentages and analyzed by the chi-square test. The Kaplan–Meier survival curve was used to compare the overall survival between the initial dialysis modalities, and the significance of the difference was tested by the log-rank method.

Subgroup analyses were performed with respect to age. Previous studies suggested that patients undergoing HD and PD have different prognosis in different age subgroups. Therefore, we used Cox regression model to confirm the interactive effect of age and dialysis type. Further analysis of the interaction effects suggested that the age between 60 and 70 years may be the cutoff point for the difference in the prognosis of different dialysis methods. We found that in the subgroups of ≤60-year-old, 60–70-year-old, and > 70-year-old, the type of dialysis had a significant effect on the prognosis.

Cox regression model was used to performed multivariate analysis (considering age, gender, primary kidney disease, and comorbidities). The covariates of multivariate regression were also determined based on clinical experience and previous studies, included sex, age at dialysis initiation, diabetes mellitus, cerebrovascular disease, congestive heart failure, chronic pulmonary disease, cardiovascular diseases, CCI, primary disease of renal failure (glomerulonephritis or polycystic kidney disease), body mass index, plasma albumin, cholesterol, and hemoglobin.

As a retrospective study, significant differences were detected in the baseline status, and hence, we used PSM to reduce these in different age groups by applying the MatchIt package in the R language for PSM at a ratio of 1:1. The matching method was nearest neighbor match. The characteristics used in PSM were the same as the variables in the multivariate Cox regression model.

Patients of different ages were grouped. Then single factor and multivariate Cox regression analysis were performed to determine the relative hazard ratio (HR) of the dialysis types, followed by PSM and multivariate Cox regression analysis to confirm the relative HR of the dialysis types.

All statistical tests were evaluated using a two-tailed 95% confidence interval (CI), and *p* < 0.05 was considered statistically significant. All statistical analyses were performed using the R language (version 3.6.0).

## Results

### Baseline characteristics of included patients

**Fig. 1:**
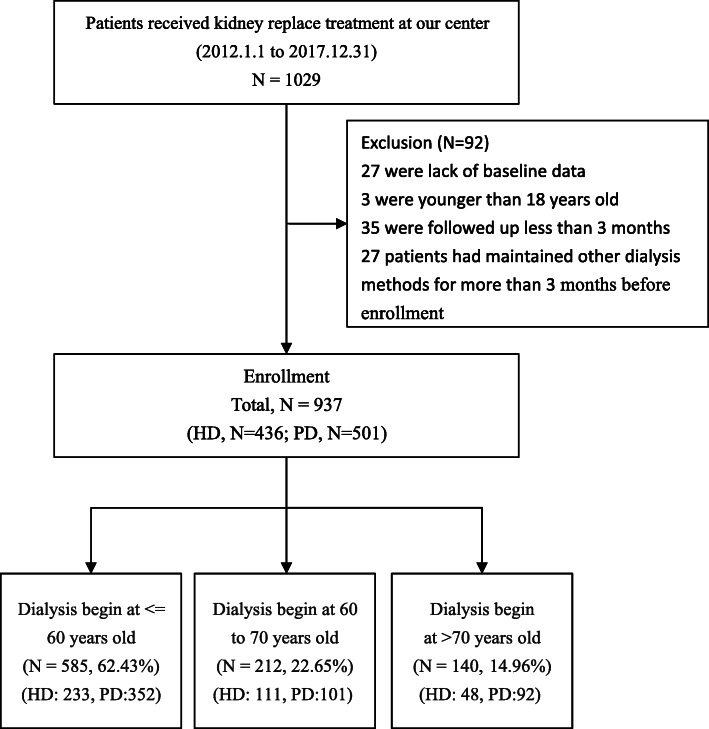
Study schematic

All patients, who started dialysis therapy between January 1, 2012 and December 31, 2017, were analyzed. A total of 1029 ESRD patients were included in this study, of whom 92 were excluded. Twenty-seven were excluded due to lack of baseline data, 3 were < 18-year-old, 35 were due to a follow-up of < 3 months, and 27 patients had maintained other dialysis methods for more than months before enrollment. Finally, there were 501 patients in the PD group and 436 in the HD group (Fig. 1).

Demographic and clinical characteristics are presented in Table 1. The mean age at dialysis start of pd. patients was younger than that of hemodialysis patients (51.69 ± 14.47 vs. 57.11 ± 15.92 years old, *p* < 0.01). The proportion of females in patients receiving PD and HD was similar (46.10% vs. 41.92%, *p* = 0.22). The follow-up duration of the HD group was longer than that of the PD group (36.98 ± 21.26 vs. 31.68 ± 18.71 months, p < 0.01). This difference was observed across all age groups.

**Table 1:** Baseline characteristics

	All Patients			Patients younger than 60 years old			Patients older than 70 years old		
	PD Group (*n* = 501)	HD Group (*n* = 436)	*p*	PD Group (*n* = 352)	HD Group (*n* = 233)	*p*	PD Group (n = 48)	HD Group (*n* = 92)	*p*
Demographic data									
Female(n)	210(41.92%)	201(46.10%)	0.22	151 (42.90%)	90 (38.63%)	0.35	19 (39.58%)	57 (61.96%)	0.02
Age at dialysis initiation (years)	51.69 ± 14.47	57.11 ± 15.92	0.00	44.55 ± 10.58	45.26 ± 11.29	0.44	75.77 ± 3.66	77.49 ± 4.49	0.02
Body mass index	22.60 ± 3.91	23.13 ± 4.30	0.05	22.34 ± 3.55	23.01 ± 4.60	0.06	22.75 ± 3.07	23.27 ± 4.13	0.40
Duration of follow up (months)	31.68 ± 18.71	36.98 ± 21.26	0.00	32.80 ± 19.44	38.28 ± 22.14	0.00	26.16 ± 15.48	34.86 ± 19.30	0.00
Kidney primary disease									
Diabetic nephropathy(n)	138 (27.54%)	116 (26.61%)	0.80	81 (23.01%)	57 (24.46%)	0.76	21 (43.75%)	26 (28.26%)	0.10
Glomerulus nephritis(n)	255 (50.90%)	136 (31.19%)	0.00	198 (56.25%)	100 (42.92%)	0.00	10 (20.83%)	15 (16.30%)	0.67
Polycystic kidney(n)	3 (0.60%)	21 (4.82%)	0.00	0 (0.00%)	14 (6.01%)	0.00	1 (2.08%)	0 (0.00%)	0.74
Obstructive nephropathy(n)	36 (7.19%)	21 (4.82%)	0.17	23 (6.53%)	9 (3.86%)	0.23	6 (12.50%)	3 (3.26%)	0.08
Other or unknow(n)	69 (13.77%)	142 (32.57%)	0.00	50 (14.20%)	53 (22.75%)	0.01	10 (20.83%)	48 (52.17%)	0.00
Comorbidities									
Charlson Comorbidities Index (CCI)	5.07 ± 2.25	5.87 ± 2.47	0.00	4.11 ± 1.69	4.36 ± 1.96	0.10	8.50 ± 1.49	8.41 ± 1.45	0.74
Diabetes(n)	164 (32.73%)	170 (38.99%)	0.05	92 (26.14%)	73 (31.33%)	0.20	26 (54.17%)	49 (53.26%)	0.94
Cardiovascular disease(n)	62 (12.38%)	64 (14.68%)	0.35	24 (6.82%)	18 (7.73%)	0.80	16 (33.33%)	27 (29.35%)	0.77
Congestive heart failure(n)	120 (23.95%)	155 (35.55%)	0.00	81 (23.01%)	63 (27.04%)	0.31	21 (43.75%)	46 (50.00%)	0.60
Cerebrovascular disease(n)	54 (10.78%)	88 (20.18%)	0.00	16 (4.55%)	30 (12.88%)	0.00	15 (31.25%)	36 (39.13%)	0.46
Chronic pulmonary disease(n)	30 (5.99%)	32 (7.34%)	0.48	16 (4.55%)	13 (5.58%)	0.71	8 (16.67%)	12 (13.04%)	0.74
Laboratory tests									
Serum Urea (mmol/l)	25.35 ± 11.77	22.26 ± 11.31	0.00	26.09 ± 12.32	22.65 ± 11.56	0.00	23.33 ± 9.84	19.12 ± 9.04	0.01
Serum creatinine (umol/l)	860.59 ± 283.38	745.81 ± 319.76	0.00	899.14 ± 283.23	804.08 ± 332.03	0.00	715.11 ± 214.78	582.44 ± 268.22	0.00
Triglyceride (mmol/L)	1.34 ± 0.79	1.43 ± 1.04	0.15	1.34 ± 0.74	1.40 ± 0.80	0.36	1.12 ± 0.70	1.35 ± 0.79	0.09
Cholesterol (mmol/L)	4.54 ± 1.35	4.45 ± 1.28	0.31	4.59 ± 1.40	4.42 ± 1.26	0.15	4.37 ± 1.35	4.39 ± 1.08	0.93
Plasma albumin(g/L)	34.87 ± 5.13	35.45 ± 5.57	0.10	34.99 ± 5.24	35.83 ± 5.97	0.08	34.09 ± 4.90	35.89 ± 4.43	0.03
Prealbumin(g/L)	316.75 ± 85.99	278.91 ± 91.84	0.00	329.16 ± 84.76	295.88 ± 97.73	0.00	242.97 ± 73.70	252.48 ± 76.24	0.48
Hemoglobin(g/L)	82.36 ± 18.67	86.66 ± 20.85	0.00	81.52 ± 18.63	87.30 ± 21.23	0.00	83.42 ± 19.30	87.00 ± 21.52	0.33

*PD* Peritoneal dialysis, *HD* Hemodialysis

In terms of primary kidney disease, diabetic nephropathy and obstructive nephropathy were similar in the two groups. In terms of primary renal diseases, glomerulonephritis accounted for a high proportion in PD group. In the HD group, polycystic kidney occupies a higher proportion. At dialysis initiation, diabetes mellitus, congestive heart failure, and cerebrovascular disease were common among the HD patients, who also presented a high CCI value. In terms of lab tests, patients who started dialysis with HD had higher triglyceride, plasma albumin, and hemoglobin, and lower serum urea, serum creatinine, cholesterol, and prealbumin than those who started dialysis with PD. Similar differences were noted in the ≤60-year-old group; however, it was not obvious in the > 70-year-old group (Table 1).

### Comparison of survival rates between the PD and HD groups

#### Overall survival

**Fig. 2:**
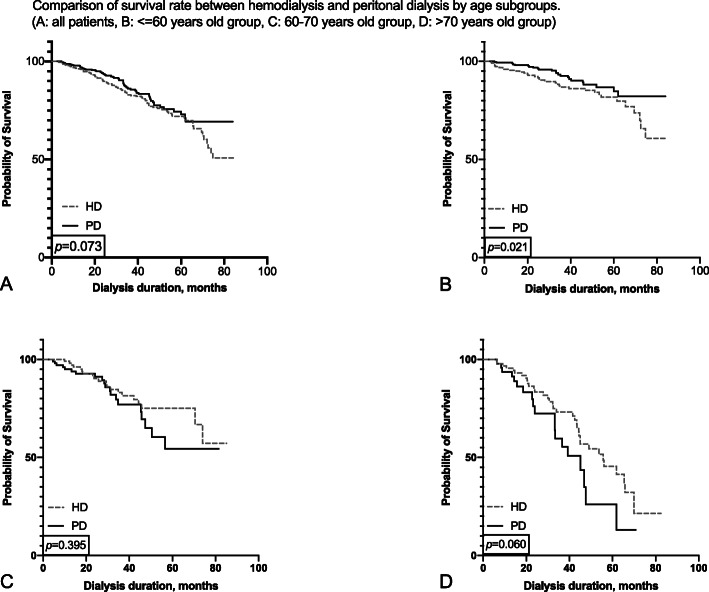
Comparison of survival rate between hemodialysis and peritonal dialysis by age subgroups. (**a**: all patients, **b**: <=60 years old group, **c**: 60–70 years old group, **d**: > 70 years old group)

The 1-, 3-, and 5-year survival rates were 98.1, 86.7, and 73.4%, respectively, in the PD group and 96.4, 83.0, and 71.9%, respectively. in the HD group. The Kaplan–Meier survival analysis showed that the overall prognosis of the two dialysis methods varied, but not significantly (*p* = 0.073, Fig. 2a).

#### Subgroup analyses by age

Cox regression analysis indicated that age was a risk factor for all-cause mortality in patients (Table 2). Previous studies from Asia have suggested that dialysis methods at different ages influence all-cause mortality. Thus, we performed a subgroup analysis based on age.

**Table 2:** Cohort outcomes

	All-cause mortality	Person-years	Mortality rate (dead/1000person-years)	Mortality rate ratio (95%,CI)
All-Patients				
HD (n = 436)	90	1325.35	67.91	1.45 (1.04–2.04)
PD (n = 501)	61	1304.52	46.76	
Age at dialysis initiation <=60				
HD (n = 233)	35	733.01	47.75	1.97 (1.13–3.49)
PD (n = 352)	23	949.10	24.23	
Age at dialysis initiation 60–70				
HD (*n* = 111)	20	328.73	60.84	0.81 (0.41–1.60)
PD (*n* = 101)	19	252.23	75.33	
Age at dialysis initiation > 70				
HD (n = 92)	35	263.60	132.77	0.72 (0.40–1.33)
PD (n = 48)	19	103.20	184.11	

*PD* Peritoneal dialysis, *HD* Hemodialysis, *95% CI* 95% confidence interval

According to the interaction effect analysis, all patients were divided into three groups base on the age at dialysis initiation (≤60-year-old, 60–70-year-old, and > 70-year-old). In the above age groups, the all-cause mortality rate ratio of HD to PD was 1.97 (95% confidence interval (CI):1.13–3.49), 0.81 (95% CI: 0.41–1.60), and 0.72 (95% CI: 0.40–1.33), respectively (Table 2).

In the ≤60-year-old subgroup, the 1-, 3-, and 5-year survival rates of the PD group were 99.4, 93.2, and 85.2%, while those of the HD group were 95.5, 87.0, and 81.8%, respectively. The Kaplan–Meier survival analysis showed the survival rate of PD was significantly higher than that of HD (*p* = 0.021, Fig. 2b). In the > 70-year-old subgroup, the 1-, 3-, and 5-year survival rates of PD group were 93.5, 59.4, and 26.0% and that of HD group were 95.5, 73.2, and 45.5%, respectively, indicating that HD had a better prognosis in this age group, but did not reach statistical significance (*p* = 0.060, Fig. 2d). On the other hand, patients in 60–70-year-old group did not show a significant difference in survival rate between HD and PD (Fig. 2c).

#### Factors associated with survival in dialysis patients

Observing the Kaplan–Meier survival curve, the prognosis of different dialysis methods in the two subgroups ≤60-year-old and > 70-year-old might be the opposite. Therefore, we performed Cox regression model analysis on these two subgroups. Considering the difference in the baseline status between the two groups, we used Cox regression model analysis again after PSM to validate our results. After PSM, the sample sizes of ≤60-year-old group changed from 233 in the HD group and 352 in the PD group to 226 in both groups, the sample sizes of > 70-year-old group changed from 92 in the HD group and 48 in the PD group to 42 in both groups.

In the ≤60-year-old subgroup, the univariate Cox regression model suggested that age at dialysis initiation, diabetes, CCI, cardiovascular disease, total cholesterol, and HD were risk factors for all-cause mortality. Cardiovascular disease, total cholesterol, and HD were still risk factors in the multivariable-adjusted model. The cholesterol and HD remained risk factors for multivariate Cox regression after using propensity scores to eliminate the differences in baseline characteristics. The HR of HD increased from 1.96 (95% CI: 1.13–3.41) to 2.26 (95% CI: 1.17–4.35). In the > 70-year-old subgroup, the univariate Cox regression model did not find any risk factors for all-cause mortality, the multivariate Cox regression model suggested HD was a protective factor. After using the PSM to eliminate the differences in baseline characteristics, HD remained a protective factor for multivariate Cox regression. The HR of HD decreased from 0.46 (95% CI: 0.23–0.91) to 0.33 (95% CI: 0.14–0.79) (Table 3).

**Table 3:** Risk factors for mortality assessed by univariate and multivariate Cox regression model

	Patients younger than 60 years old PD (n = 352), HD (*n* = 233)			Patients older than 70 years old PD (*n* = 48), HD (*n* = 92)		
	Univariate HR (95% CI)	Multivariate^a^ HR (95% CI)	PSM^b^-Multivariate^a^ PD and HD (*n* = 226) HR (95% CI)	Univariate HR (95% CI)	Multivariate^a^ HR (95% CI)	PSM^b^-Multivariate^a^ PD and HD (*n* = 42) HR (95% CI)
Age at dialysis initiation (per 1 year)	**1.03 (1.00–1.06)**	1.02 (0.99–1.05)	1.02 (0.99–1.06)	1.05 (0.98–1.11)	1.05 (0.98–1.13)	1.07 (0.96–1.18)
Sex (female vs male)	0.58 (0.33–1.00)	**0.53 (0.29–0.95)**	0.58 (0.30–1.13)	0.83 (0.49–1.42)	0.90 (0.49–1.63)	1.14 (0.47–2.75)
Diabetes(n)	**1.89 (1.12–3.20)**	1.14 (0.62–2.11)	1.26 (0.46–3.44)	1.00 (0.58–1.70)	1.13 (0.61–2.09)	1.77 (0.61–5.17)
Hypertension(n)	1.68 (0.67–4.20)	1.33 (0.50–3.52)	1.44 (0.43–4.86)	1.18 (0.16–8.56)	1.52 (0.19–12.24)	0.95 (0.10–8.92)
Charlson Comorbidities Index (CCI)	**1.23 (1.07–1.41)**	0.93 (0.64–1.36)	0.63 (0.24–1.66)	1.02 (0.82–1.25)	0.90 (0.61–1.32)	1.06 (0.64–1.75)
Congestive heart failure(n)	1.51 (0.87–2.61)	1.11 (0.61–2.00)	1.22 (0.56–2.65)	1.08 (0.63–1.85)	0.99 (0.52–1.85)	1.35 (0.49–3.71)
Cerebrovascular disease(n)	1.72 (0.78–3.80)	1.21 (0.52–2.81)	1.24 (0.42–3.65)	1.04 (0.60–1.79)	0.83 (0.44–1.57)	0.56 (0.19–1.63)
Chronic pulmonary disease(n)	1.42 (0.51–3.93)	1.07 (0.38–3.02)	0.93 (0.26–3.27)	1.83 (0.96–3.48)	1.68 (0.81–3.50)	1.00 (0.34–2.96)
Cardiovascular disease(n)	**2.87 (1.41–5.86)**	**2.56 (1.16–5.66)**	1.79 (0.71–4.51)	1.29 (0.73–2.28)	1.44 (0.70–2.95)	**3.61 (1.31–10.00)**
Body mass index (per 1 kg/m2)	1.04 (0.98–1.11)	1.01 (0.94–1.08)	1.02 (0.95–1.10)	0.95 (0.88–1.02)	0.96 (0.88–1.04)	0.88 (0.77–1.01)
Hemoglobin (per 1 g/L)	1.01 (0.99–1.02)	1.00 (0.99–1.02)	1.00 (0.99–1.02)	1.00 (0.98–1.01)	1.00 (0.98–1.01)	1.01 (0.99–1.03)
Plasma albumin (per 1 g/L)	0.97 (0.93–1.02)	0.98 (0.94–1.04)	0.99 (0.93–1.04)	0.96 (0.91–1.02)	0.96 (0.90–1.02)	1.02 (0.92–1.13)
Triglyceride (per 1 mmol/L)	1.00 (0.97–1.03)	1.03 (0.98–1.08)	1.20 (0.78–1.84)	0.98 (0.95–1.02)	1.00 (0.94–1.05)	1.41 (0.63–3.12)
Cholesterol (per 1 mmol/L)	**1.31 (1.12–1.54)**	**1.37 (1.16–1.63)**	**1.27 (1.02–1.58)**	1.04 (0.79–1.36)	1.07 (0.79–1.46)	1.13 (0.73–1.75)
Dialysis Methods (HD vs. PD)	**1.85 (1.09–3.14)**	**1.96 (1.13–3.41)**	**2.26 (1.17–4.35)**	0.58 (0.33–1.03)	**0.46 (0.23–0.91)**	**0.33 (0.14–0.79)**

*PSM* Propensity score match, *HR* Hazard ratio, *95% CI* 95% confidence interval, *PD* Peritoneal dialysis, *HD* Hemodialysis

^a^Variables adjusted in multivariate Cox model for both subgroups: sex, age at dialysis initiation, diabetes mellitus, cerebrovascular disease, congestive heart failure, chronic pulmonary disease, cardiovascular diseases, CCI, primary disease of renal failure (glomerulonephritis or polycystic kidney disease), body mass index, plasma albumin, cholesterol, and hemoglobin.

^b^The characteristics used in PSM included: sex, age at dialysis initiation, diabetes mellitus, cerebrovascular disease, congestive heart failure, chronic pulmonary disease, cardiovascular diseases, CCI, primary disease of renal failure (glomerulonephritis or polycystic kidney disease), body mass index, plasma albumin, cholesterol, and hemoglobin.

#### Short- and long-term survival

**Fig. 3:**
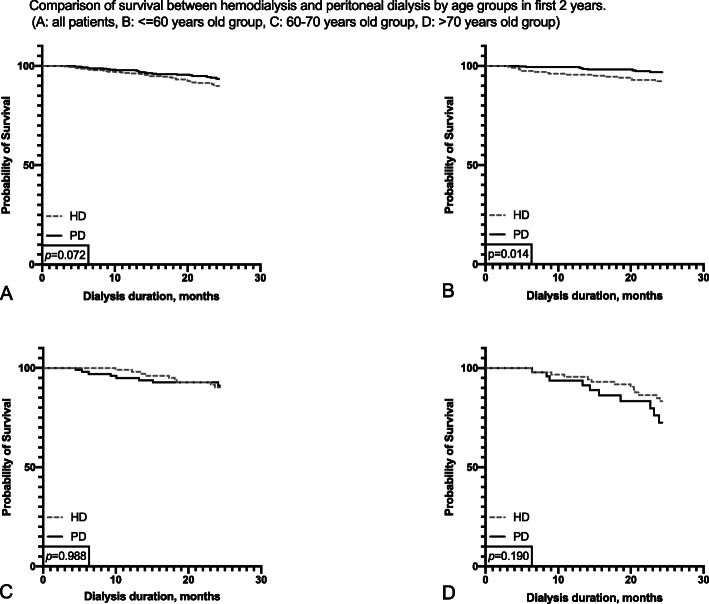
Comparison of survival between hemodialysis and peritoneal dialysis by age groups in first 2 years. (**a**: all patients, **b**: <=60 years old group, **c**: 60–70 years old group, **d**: > 70 years old group)

**Fig. 4:**
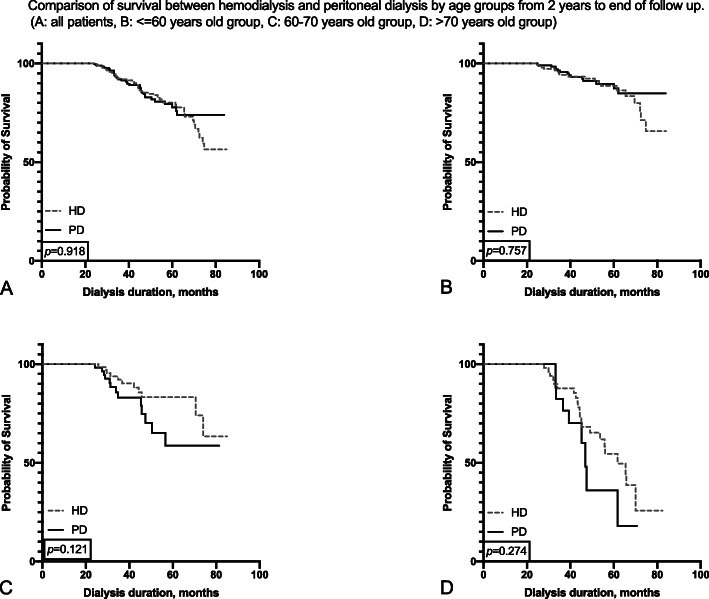
Comparison of survival between hemodialysis and peritoneal dialysis by age groups from 2 years to end of follow up. (**a**: all patients, **b**: <=60 years old group, **c**: 60–70 years old group, **d**: > 70 years old group)

We found that within the first 2 years after dialysis initiation, PD patients in the entire population had a tendency to survive better, but the benefit failed to reach statistical significance. In < 60-year-old subgroup, PD patients had survival advantage reached statistical significance (*p* = 0.014, Fig. 3b), but this advantage was not found in other subgroups (Fig. 3). After the second year of follow-up, no differences in survival were found between the two dialysis methods (Fig. 4).

## Discussion

In this study, we compared HD and PD and found that the overall survival of PD was better than that of HD in the ≤60-year-old group. This difference was still significant after the adjustment for a variety of confounding factors and PSM. The advantage of PD in young patients was first discovered in the current study. On the other hand, in the analysis of the univariate Cox regression model, the > 70-year-old group did not prove the protective effect of HD. However, in the multivariate Cox regression model and the PSM multivariate Cox regression model, the protective effect of HD is statistically significant. Our findings suggested that PD could be a better choice for younger patients (≤60-year-old), and HD could be a better choice for older patients (> 70-year-old).

We compared 501 HD patients and 436 PD patients and found that PD patients had better survival rate than HD patients (1-, 3-, and 5-year survival rates were 98.1, 96.3, and 86.3% vs. 82.7, 72.5, and 71.1%), albeit not significantly. The current result concurred with the observational study in Beijing, China [13]. A study with 871 ESRD patients in Singapore showed that patients who initiated dialysis with HD experienced better survival outcomes than those who initiated dialysis with PD [8]. This phenomenon might be related to the older age at dialysis start of PD patients (mean age 58.2-year-old for HD vs. 64.3-year-old for PD). Conversely, in this cohort, patients with PD were younger (mean age 57.11-year-old for HD vs. 51.69-year-old for PD). Thus, the age at which dialysis was started might be the key to patient survival. A 2008 study in Taiwan suggested that PD has a better prognosis in non-diabetic patients < 55-year-old, while in other subgroups, the prognosis is similar [12]. Another study in South Korea demonstrated that the survival outcomes of these two dialysis modalities are similar for patients < 55-year-old, whereas HD is superior to PD for the elderly [7]. A study in Taiwan suggested that the overall survival time of PD patients has improved in recent years [12]. This phenomenon might be related to the continuous improvement of PD training methods and new dialysate applications [15]. Therefore, we speculate that the survival time of PD patients can be prolonged through better chronic disease management and peritoneal dialysis technology. Previous comparative studies in the United States [16], Australia and New Zealand [17] had found that peritoneal dialysis had a survival advantage during the first year or two of dialysis [18]. According to our data, we found that within the first 1–2 years after dialysis initiation, PD in the entire population had a tendency to survive better, but failed to reach statistical significance. In < 60-year-old subgroup, PD had survival advantages, but the other subgroups did not find similar advantages. After the second year of follow-up, no differences in survival were found between the two dialysis methods.

Regarding the risk factors associated with survival, age, diabetes [13, 19–21], serum albumin [19], BMI [13], and cardiovascular disease [21, 22] were not consistent with our results. Importantly, age is one of the major factors affecting survival in dialysis patients, which is consistent with our findings. Therefore, we performed subgroup analysis by age. Also, we found that the risk factors were not completely similar in different age subgroups (≤60 and > 70-year-old). In the younger subgroup, CCI, cardiovascular disease, total cholesterol, and HD were risk factors of all-cause mortality. In the older subgroup, PD was risk factor.

The previous history of cardiovascular disease and diabetes mellitus was associated with left ventricular ejection fraction in PD patients [23]. Hypercholesterolemia was associated with increased mortality in HD patients without myocardial infarction/cardiovascular disease [24], while high pulmonary artery systolic pressure predicted the development of right ventricular dysfunction, which portends a poor prognosis [24]. The above three studies suggested that heart failure might be a main cause of mortality in young subgroups. PD might confer a survival advantage to young and healthier patients due to better preservation of residual renal function as compared to those undergoing HD [25]. The problem of ultrafiltration in the later period of PD could be solved by self-management and the use of icodextrin [25]. Therefore, the advantages of PD in younger subgroup might be due to the advantages of PD in the treatment of heart failure. Therefore, we performed a sensitivity analysis which includes patients with congestive heart failure only. The advantages of PD in young patients remained, which proved our speculation. However, the efficacy of HD and PD in patients with heart failure was still controversial. Further researches needed to be conducted.

CCI was originally used to estimate the 1-year mortality rate of hospitalized patients [26]. The condition of the population in this article is relatively mild and the follow-up time is longer, so CCI may not accurately reflect the impact of comorbidities on the prognosis. Hemmelgarn et al’s study had shown that congestive heart failure, chronic pulmonary disease, and cardiovascular disease have a greater impact on the mortality of ESRD patients than other comorbidities [27]. Therefore, these comorbidities were added in to the model. However, due to the correlation between CCI and these comorbidities, the original model may have multicollinearity problems. For this reason, we calculated the variance inflation factor of the original model. We found mild multicollinearity that is acceptable. We also tried the model using CCI and the model using comorbidities separately. We found that the survival advantage of PD in the ≤60-year-old subgroup remained. For the > 70-year-old subgroup, the advantage of HD in the multivariate Cox regression remained, but the advantage in the Cox regression model after PSM disappeared. Generally, it may not be appropriate to use both comorbidities and CCI as covariates in one model, considering that the degree of multicollinearity is acceptable, the results obtained by different models differ little, and we still maintain the original model.

People who are on PD often have a more flexible treatment schedule than people on in-center dialysis. Choosing to do PD instead of in-center hemodialysis may enable patients to keep their regular schedule for work, school or other activities. Younger ESRD patients took advantage of this freedom to return to work and thus received better financial support, which in turn prolonged their survival. Older ESRD patients had poorer self-care ability and needed to rely on the assistance of family members and medical institutions. The in-center dialysis provides clinical assessment twice or thrice a week by clinicians and nurses, therefore, it is rather beneficial to older ESRD patients. PD could cause significant protein-energy loss [28], decreased nutritional intake of older patients might be the cause of a shortened life span. Although this research could not prove the above points, it was speculated as a reasonable explanation and a direction for future research.

This study was a single-center study, which had a longer follow-up time and a larger number of cases than other similar studies in our country [13]. For the first time, the survival of PD and HD patients was compared in southern China. Our study provides insight for the selection of dialysis methods for ESRD patients in the city where the research center is located.

### Limitations

The retrospective medical record data might deviate from the judgment of the primary disease of renal failure. Herein, only baseline laboratory test results were recorded, which might alter during dialysis treatment. Therefore, hemoglobin, plasma albumin, calcium, and phosphorus could not reflect the situation of patients in the treatment process. This study does not include information about patient, professional status, education level, and medical insurance, and therefore cannot assess the impact of financial support on patient survival. In addition, no specific cause of death was investigated in this study.

The high demand for kidney replacement therapy and the shortage of HD equipment have encouraged the clinicians and patients to prefer PD to HD in recent years, which would lead to significant patient selection bias. Our center is located in Guangzhou, one of China’s most advanced cities in economy and medical technology, therefore, the population in this study do not represent other regions of China. Above situation may be the reason for the significantly higher overall survival rate of patients in this study. When extrapolating the conclusions of this study, the factors of economic and technical support should be fully considered.

## Conclusions

For younger ESRD patients (≤60-year-old), the 1-, 3-, and 5-year survival rates of PD were higher than those of HD. For older ESRD patients (> 70-year-old), the 1-, 3-, and 5-year survival rates of PD were lower than those of HD. Therefore, the results suggested that PD may be a better choice for younger ESRD patients, and HD for the older patients.

## Data Availability

The datasets used and/or analyzed during the current study are available from the corresponding author on reasonable request and with permission of Guangdong Provincial Hospital of Chinese Medicine.

## Abbreviations

ESRD: End-stage renal disease
HD: Hemodialysis
PD: Peritoneal dialysis
RCT: Randomized controlled trial
CAPD: Continuous ambulatory peritoneal dialysis
ICD: International Classification of Diseases
CCI: Charlson comorbidities index
SD: Standard deviation
PSM: Propensity score matching
HR: Hazard ratio
CI: Confidence interval
